# A novel mutation in the *UBAP1* gene causing hereditary spastic paraplegia: A case report and overview of the genotype-phenotype correlation

**DOI:** 10.3389/fgene.2022.936292

**Published:** 2022-07-14

**Authors:** Peiqiang Li, Xiande Huang, Senmao Chai, Dalin Zhu, Huirong Huang, Fengdie Ma, Shasha Zhang, Xiaodong Xie

**Affiliations:** ^1^ Institute of Genetics, School of Basic Medical Sciences, Lanzhou University, Lanzhou, China; ^2^ Department of Urology, Gansu Provincial Hospital, Lanzhou, China; ^3^ Medical Imaging Center, Gansu Province Maternal and Child-care Hospital, Lanzhou, China; ^4^ Department of Respiratory Medicine, Lanzhou University Second Hospital, Lanzhou, China

**Keywords:** case report, UBAP1, hereditary spastic paraplegia, whole-exome sequencing, novel mutation

## Abstract

Hereditary Spastic Paraplegia (HSP) is considered to be one of the common neurodegenerative diseases with marked genetic heterogeneity. Recently, the mutations in ubiquitin-associated protein 1 (*UBAP1*) have been described in patients with HSP, known as spastic paraplegias 80 (SPG80). Here, we reported a Chinese HSP family presenting a frameshift mutation in the *UBAP1* gene leading to complex HSP. Their clinical features encompassed spastic paraparetic gait, exaggerated patellar tendon reflexes, bilateral Babinski signs, and hyperactive Achilles tendon reflex. The proband also had severe urinary incontinence and a dermoid cyst at the lumbar 4–5 spinal cord, which rarely occurs in HSP patients. Following whole-exome sequencing, a novel heterozygous mutation (c.437dupG, NM_016,525) was identified in the *UBAP1* that segregated with the family’s phenotype and resulted in truncating UBAP1 protein (p.Ser146ArgfsTer13). Moreover, we reviewed the genotypes of *UBAP1* and the phenotypic variability in 90 HSP patients reported in the literature. We found that the age of onset in UBAP1-related patients was juvenile, and there were population differences in the age of onset. The main complications were lower extremity spasticity, hyperreflexia, and the Babinski sign. Exon 4 of UBAP1 was identified as a mutation hotspot region. Our study expands the knowledge of UBAP1 mutations, which will aid in HSP patient counseling. Further molecular biological research is needed to explore the genotype-phenotype correlations of UBAP1-related HSP.

## Introduction

Hereditary spastic paraplegia (HSP) refers to a serious monogenic neurodegenerative disorder characterized by progressive lower limb spasticity, with a prevalence of 2–5 cases per 100,000 individuals worldwide (Boutry et al., 2019). HSP is classified into pure or complicated forms. The main syndromes of pure HSP are limited to the lower limbs, whereas the complex form is accompanied by additional neurologic or non-neurological impairment signs, such as cognitive disorders, ataxia, seizures, neuropathy, and so on (de Souza et al., 2016). In addition, HSP presents significant genetic heterogeneity, and more than 80 spastic paraplegia genes (SPG) or loci have been identified (Shribman et al., 2019).

Ubiquitin-associated protein 1(UBAP1) acts as the subunit of the endosomal sorting complex required for transport (ESCRT) -I to bind the ubiquitin-conjugated membrane proteins into the intraluminal vesicles of multivesicular bodies (MVB) (Stefani et al., 2011; Agromayor et al., 2012). It has been reported that *UBAP1* was associated with frontotemporal lobar degeneration (FTLD) (Rollinson et al., 2009), and aberrant methylation changes of *UBAP1* were associated with gouty inflammation, (Tseng et al., 2020). It was also found that UBAP1 interacted with Toll/interleukin 1 receptor (TIR) domain-containing protein of *Pseudomonas aeruginosa*, and played a major role in virulence of bacterial pathogens Imbert et al. (2017). Recently, the mutations in *UBAP1* were found to cause the pure or complex HSP with autosomal dominant inheritance (Farazi Fard et al., 2019; Lin et al., 2019; Nan et al., 2019; Bourinaris et al., 2020; Gu et al., 2020; Wang et al., 2020; Bian et al., 2021). Therefore, *UBAP1* was known as the pathogenic gene of spastic paraplegia-80 (SPG80, OMIM No. 618418).

We here reported the clinical and genetic findings in UBAP1-related HSP patients in the HSP family from Northwest China. These patients presented with complex or pure form HSP. However, in addition to urinary incontinence, the proband (V-1) was found to have dermoid cysts at the lumbar 4–5 level of the spinal cord, which rarely occurs in HSP patients. Further sequencing revealed that patients (III-2, IV-2, IV-3 and V-1) in this family had a novel mutation in *UBAP1.* For delineating the phenotype-genotype correlation, we reviewed all published UBAP1-related HSP cases. Finally, a total of 90 patients, who carried 20 various *UBAP1* mutations, were identified in the literature. We further discussed the correlations between phenotypes and genotypes that would improve the prediction of clinical phenotypes.

## Methods

### Subjects

**FIGURE 1:**
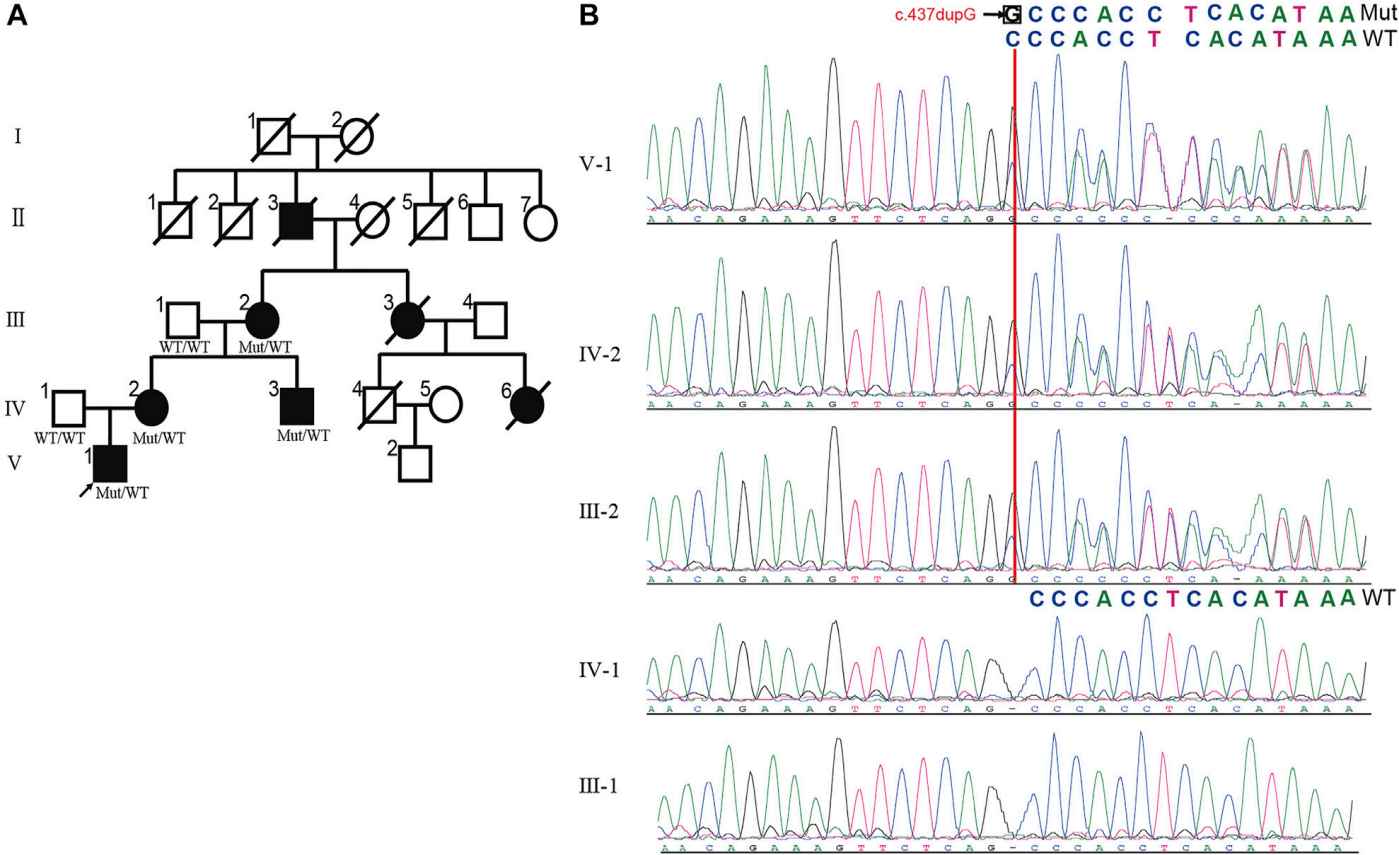
The pedigree of HSP with UBAP1 mutation. **(A)** The inheritance pattern in this family showed classic autosomal dominant inheritance. HSP patients are marked by black symbols. An arrow indicates the proband. The genotypes of all available family members are displayed with c.279delG mutation and wild typeallele. **(B)** Alignment of Sanger sequencing results showed c.437dupG induced mRNA frameshift of UBAP1 gene. The c.437dupG (red arrow) was detected in patients, but not in healthy members, which showed the mutation is the co-segregation with the manifestations in the family. Notes:Mut, Mutation; WT, wild type.

The pedigree with HSP was from the Northwest of China and was presented in Figure 1A. There were 7 affected individuals and no consanguineous relationship in this family (Figure1A). The proband was a 14 years old male, who was admitted to a local hospital with urinary incontinence and progressive gait imbalance for 4 years. The other three patients (IV-2, IV-3, and III-2) also suffered from the feature of prominent lower-extremity spasticity. After obtaining written informed consent, all available family members were systematically examined by the same experienced neurologist.

### Exome sequencing and data analysis

Blood samples from the proband (V-1), his parents (IV-1 and IV-2), his uncle (IV-3), and his grandparents (III-1 and III-2) were collected (Figure1A). The whole-exome sequencing (WES) was performed on V-1, IV-1, and III-2. Genomic DNA preparation and sequencing were carried out as previously described (Ma et al., 2019). Sequencing was performed at BGI (Shenzhen, China). The captured libraries were sequenced on Illumina HiSeq platforms with 150bp pair-end reads. The sequencing reads were aligned to human reference genome (UCSC hg19) with BWA2 (v0.7.15). Genotypes were performed using an in-house bioinformatics pipeline.

Then we used ANNOVAR for functional annotation with the 1,000 Genomes Project, Exome Aggregation Consortium (ExAC), NHLBI Exome Sequencing Project (ESP), and Online Mendelian Inheritance in Man (OMIM). Variants that possibly impair the protein function, i.e., missense, nonsense, conserved splice sites, read-throughs, or small insertions/deletions, were prioritized. The predicted pathogenic functional effects of the variants were analyzed with Polyphen2, SIFT, and MutationTaster. The candidate pathogenic mutation (NM_021213, c.437dupG) was verified by Sanger sequencing in the DNA samples obtained from the family.

To predict the protein three-dimensional (3D) structure of the mutant c.437dupG (p.Ser146ArgfsTer13), we retrieved the protein structure of UBAP1 wild-type from the AlphaFold protein structure database (the AF-Q9NZ09-F1-model_v2 template, https://www.alphafold.ebi.ac.uk/). Then, the mutant protein structure was constructed by the I-TASSER tool (https://zhanglab.ccmb.med.umich.edu/I-TASSER/).

### Statistical analysis

The R software (version 3.6.3) was used to perform a single-group Meta-analysis of the age of onset, and the *I* ^2^ test was used to test the heterogeneity of the included literature. When *I* ^2^ > 50% was considered to have heterogeneity, a random-effects model was selected. The “trackViewer” package (version 1.24.2) in R was used to draw lollipop figures of *UBAP1* mutation sites.

## Case presentation

### Clinical history and examination

The non-consanguineous family of pure HSP was from North-western China. A total of seven patients in this family were involved in four generations. The family tree showed an autosomal dominant pattern of inheritance (Figure1A). The proband (V-1), aged 14, developed lower limb dyskinesia and urinary incontinence when he was 10 years old. He self-reported undergoing spinal surgery due to a dermoid cyst at the lumbosacral spinal cord at the age of 12. He was readmitted to the hospital because of the gradual worsening of urinary incontinence and abnormal gait. After a detailed neurological evaluation, the proband was found to exhibit the spastic paraparetic gait, exaggerated patellar tendon reflexes, bilateral Babinski signs, and hyperactive Achilles tendon reflex (Table1). The proband’s mother (IV-2) and uncle (IV-3) had a similar gait to him, while his grandmother (III-2) had to use crutches to walk. The two female patients also had positive abdominal reflexes but negative in the two male patients in the family (Table 1). The cognitive screening of the members of the family was normal (Table 1). None of the patients in the family exhibited other complications such as ataxia, dysphagia, dysarthria, cerebellar abnormalities, and eye movement involvement. According to the proband’s mother, the patients III-3 and IV-6 died in a car accident.

**TABLE 1:** Detailed clinical information of affected individuals in HSP patients of this family.

Patient ID	V-1	Ⅳ-2	Ⅳ-3	Ⅲ-2
Ethnicity	Chinese	Chinese	Chinese	Chinese
Gender	Male	Female	Male	Female
Age at last examination (years)	14	35	34	55
Age of onset (years)	10	12	14	12
Duration	4	23	20	43
Walking aid	-	-	−	+
Lower Limb spasticity	+/−	+/−	+/−	+/−
Lower Limb distal weakness	+	−	−	−
Lower Limb distal amyotrophy	−	−	−	−
Babinski sign	+	+	+	+
PTR (patellar tendon reflex)	+	+	+	+
Hyperactive Achilles tendon reflex	+	+	+	+
abdominal reflexes	−	+	−	+
Lower Limb deep sensory disturbances	Normal	Normal	Normal	Normal
Foot deformity	Normal	Normal	Normal	Normal
Upper Limb spasticity	Normal	Normal	Normal	Normal
Upper Limb weakness	Normal	Normal	Normal	Normal
Upper Limb sensory disturbances	−	−	−	−
Upper Limb DTR(deep tendon reflexes)	Normal	Normal	Normal	Normal
urinary incontinence	+	−	−	−
Ataxia	−	−	−	−
Brain MRI	hyperintense signal on the right corticospinal tract pathways	hyperintense signal on both corticospinal tract pathways	hyperintense signal on corticospinal tract pathways	hyperintense signal on corticospinal tract pathways
Spine MRI	Dermoid cyst from L4 to L5	Normal	Normal	Normal

+, positive; −, negative.

**FIGURE 2:**
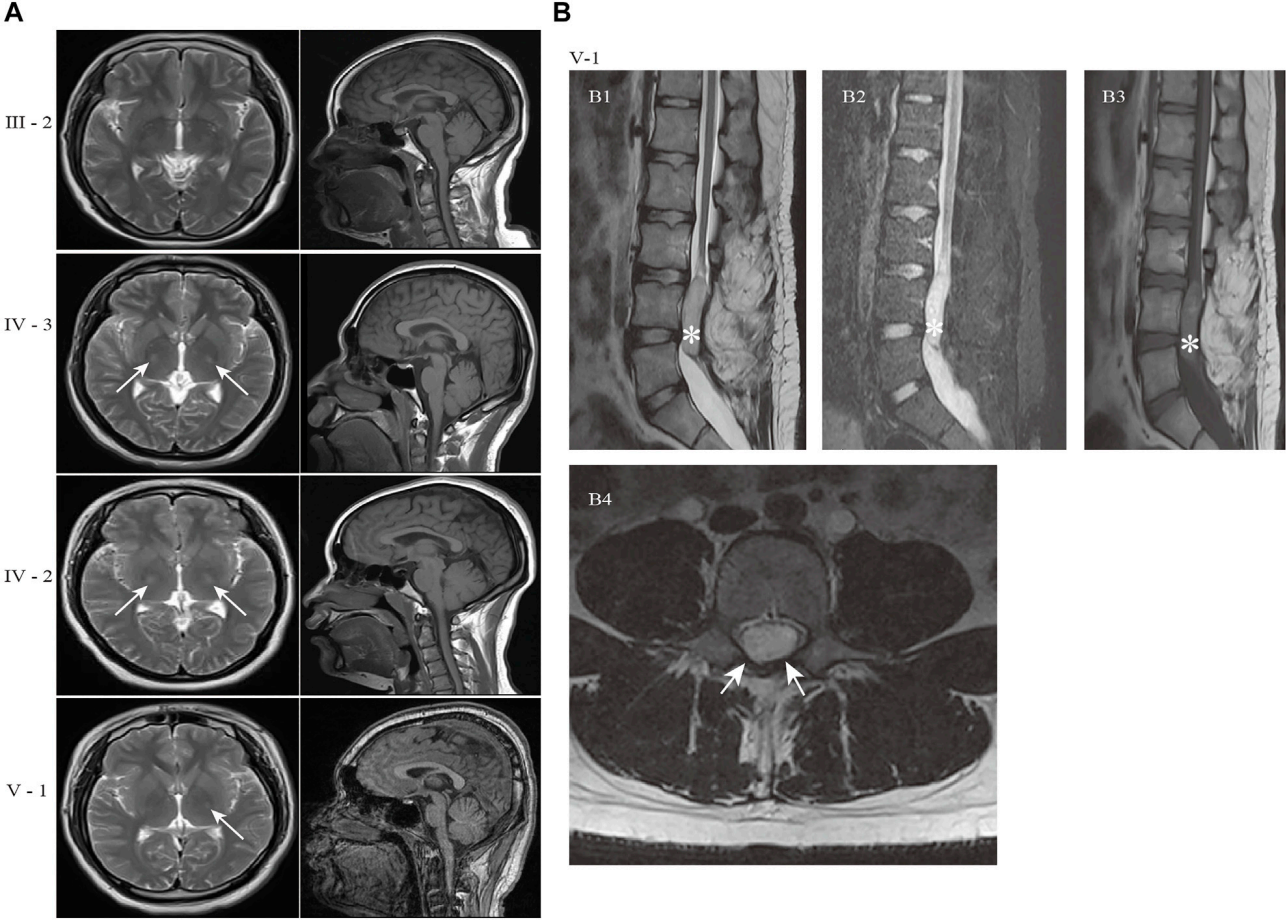
Brain and spine MRI of the patients in the HSP family. **(A)** Axial T2-weighted images and sagittal T1-weighted images of patients. The hyperintensity on the left corticospinal tract pathways were present in proband (V-1). The patient IV-2 and IV-3 showed the bilateral hyperintensities (white arrows). The subjects in this family did not have signs of corpus callosum atrophy. **(B)** MR image of proband (V-1) showed an oval space-occupying lesion with well-defined boundary in the spinal canal at the corresponding to L4-5 level (white asterisk). (B1) Sagittal T2WI; (B2) Sagittal fat-suppressed T2WI; (B3) Sagittal T1WI; (B4) Axial T2WI. The abnormal signal shadow was uniformity hyperintense signal on T2WI, while no signal reduction on fat-suppressed T2WI (B2). The shadow showed nonuniform and inhomogeneous signals on T1WI(B3). The dural sac was partially compressed in B4 (white arrows). Sagittal T2WI: T2-weighted Imaging; T1WI: T1-weighted Imaging.

Brain magnetic resonance imaging (MRI) of the proband revealed the right hyperintense signal on corticospinal tract pathways, and the patients IV-2 and IV-3 showed the bilateral hyperintensities (Figure 2A). The subjects in this family did not have signs of corpus callosum atrophy. It is worth noting that the spinal MRI of the proband revealed a dermoid cyst at the lumbar 4–5 level of the spinal cord (Figure 2B, asterisk), which was located at the area of the previous surgical resection. The lesion was hyperintense on T2WI (Figure 2B1), high signal intensity on fat-suppressed T2WI (Figure 2B2), slightly higher signals on T1WI (Figure 2B3) and the majority of the signals were uniform and well defined. The boundary of the lesion was clear and the dural sac was partially compressed (Figure 2B4). No abnormal signals changes on adjacent vertebral body. We were unable to make a further pathological analysis of this lesion.

### Identification of a novel variant in *UBAP1*

We performed WES on blood genomic DNA from the patient (V-1), his father (IV-1), and his grandmother (III-2). Only non-synonymous, splice acceptor and donor site and coding insertion/deletion (indel) variants with a minor allele frequency of less than 0.001 in East Asian (EAS) from 1,000 genomes or gnomAD database were further considered. A dominant model was assumed and 34 rare heterozygous mutations were shared by the two affected individuals but absent in the unaffected individual (Supplementary Table S1). Of the variants, we found the novel insertion mutation c.437dupG in exon4 of *UBAP1* (NM_016,525) (ubiquitin associated protein 1) as the causative variant. According to OMIM, the remaining 33 mutated genes have not been reported to be associated with the occurrence of HSP. By Sanger sequencing (Figure 1B), we confirmed that the mutation of *UBAP1* was present in other patients of the family (IV-2 and IV-3) but not in the normal individuals (III-1 and IV-1), suggesting that there is a co-segregation between the mutation and the manifestations of the patients.

**FIGURE 3:**
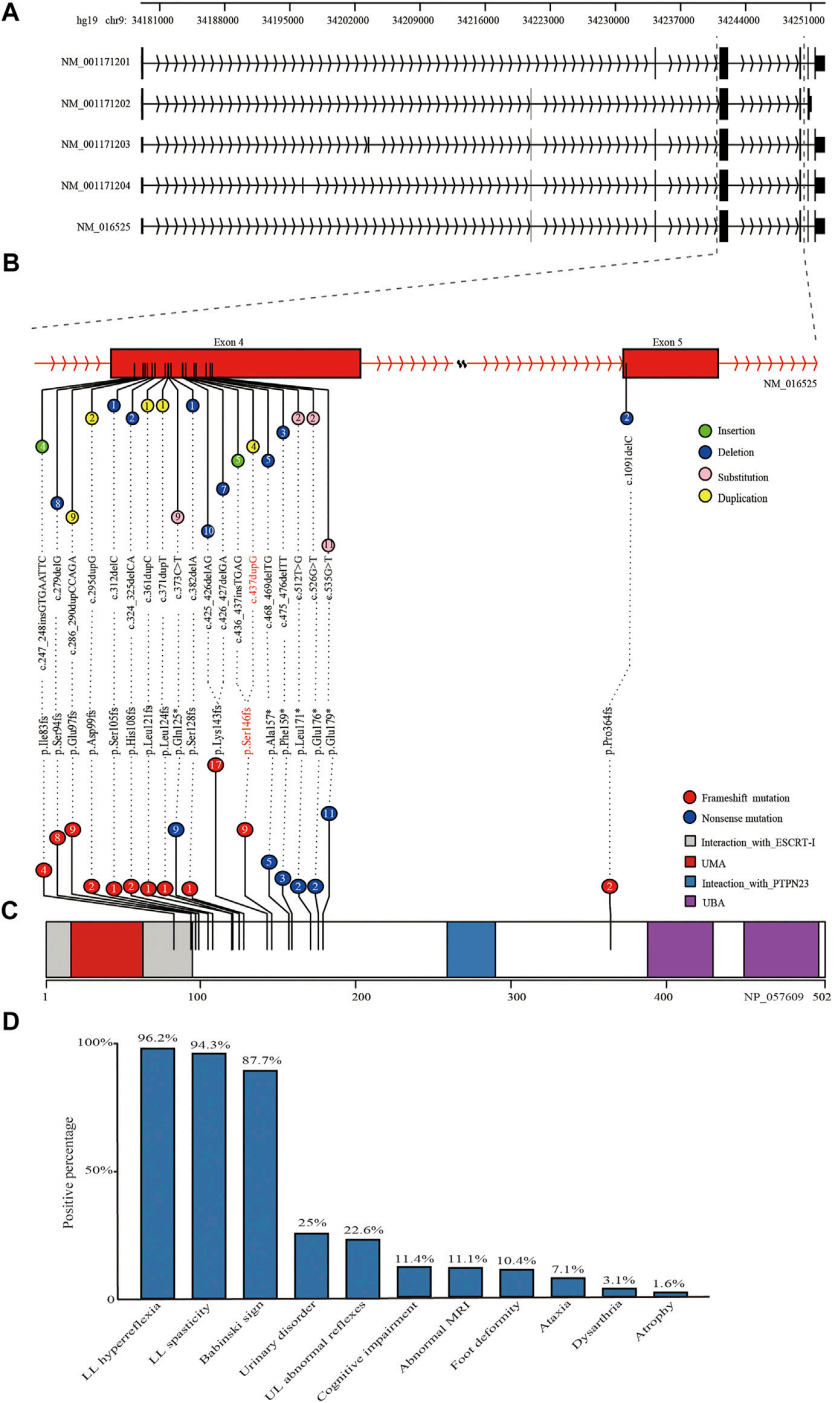
Schematic diagram of reported mutations and clinical features of UBAP1-related HSP. **(A)** The five transcripts of *UBAP1* from the database of NCBI RefSeq. All of reported mutations in *UBAP1* were displayed in the transcript NM_016,525 **(B)** and UBAP1 protein **(C)**. The numbers in lollipop represents the mutation number. The mutation found in our study was marked in red. **(D)** Phenotypic heterogeneity was observed in HSP patients with *UBAP1* mutated. The lower limb (LL) hyperreflexia and spasticity, positive Babinski sign were the main symptoms in patients with *UBAP1* mutation. Nearly one quarter of patients suffered from bladder and upper extremity muscle reflexes.

The c.437dupG was predicted to lead to a reading frame shift at position 146 and a stop codon (p.Ser146ArgfsTer13), with a concomitant 70.9% reduction in protein length (Figure3B). According to the American College of Medical Genetics and Genomics (ACMG) (Richards et al., 2015), the mutation meets multiple effect criteria making its pathogenic significance “likely pathogenic” (functional characterization-PVS1, population data-PM2, family co-segregation-PP1, in silico predictions-PP3, and family phenotypes highly specific for gene-PP4).

To analyze the structure of the truncated protein (p.Ser146ArgfsTer13), we performed three-dimensional structure predictions for the mutant. The predicted results showed that the secondary structure of the UBAP1 truncated protein was significantly changed. Comparing the structures of UBAP1 wild-type and truncated proteins, it was found that the truncated protein retains a partially similar structure to the wild-type protein only at the N-terminus (Supplementary Figure S1).

### Literature review on the phenotype and genotype characteristics of HSP caused by mutations of *UBAP1*

We used the search terms “*UBAP1”* AND “hereditary spastic paraplegia” to retrieve eight results on PubMed (last performed on 31 March 2022). Overall, nine studies, including our study, were finally included in the following analysis. We identified 35 families with HSP and screened a total of 90 HSP patients with genetically confirmed *UBAP1* mutations. Their detailed clinical features were summarized in Supplementary Table S2. The meta-analysis showed the mean age of onset was 10.71 (95%CI = 8.76–12.66). European patients [9.01 (95%CI = 5.61–12.42) in the random-effects model] were younger than Asian patients [11.66 (95%CI = 9.95–13.36) in the random-effects model] (Supplementary Figure S2). We found that female HSP patients were more reported than males, and the ratio was 1.72 (57 female vs*.* 33 male) (Supplementary Table S2). This proportion was consistent in patients of Asian and European origin, with no significant population differences. Most patients with *UBAP1* mutations had clinical symptoms consistent with pure HSP. The lower limb (LL) spasticity and hyperreflexia, positive Babinski sign were the main complications of UBAP1-related HSP (Figure 3D). In addition, abnormal bladder and upper limb (UL) abnormal muscle reflexes occur in 25% and 22.6% of patients, respectively. Only a part of the patients showed complex forms of HSP, especially the families from Germany, who carried the c. 286_290 dupCCAGA mutation, had mild to moderate symptoms of cognitive deficits (Farazi Fard et al., 2019).

At present, 20 mutations in *UBAP1* have been found in HSP patients, nineteen of which were located in exon 4 Figure 3A, and only c.1091delC was in exon 5. These variation types included insertion, duplication, and nonsense (Figure 3B). Of the mutations, the c.425_426delAG (found in ten patients) and c.426_427delGA (found in seven patients) were the most common, which affected 19.3% of patients from 11 HSP families from Asia and Europe and generated the similar premature stop codon (p.Lys143fs). The nonsense mutation c.535G > T (p.Glu179Ter) was identified in about 10.2% of patients (nine patients for one Japanese family and four United Kingdom families). In addition, both the c. 436_437insTGAG found by Farazi Fard et al. (2019) and the c.437dupG in this study were predicted to produce a similar frameshift mutation at the 146th codon (p.Ser146Metfs*14 and p. Ser146Argfs*13). All reported mutations produced the truncate UBAP1 protein and the loss of the UBA (UBAP1-MVB12-associated) domain, which were supposed to lead to dominant-negative (DN) UBAP1 proteins (Figure 3C) Lin et al. (2019).

## Discussion

In this report, we described the clinical features and genetic analyses of a pure form of the dominant HSP family from Northwest China. Finally, a novel frameshift mutation (NM_016525, c.437dupG, in a heterozygous state) in *UBAP1* was identified. Furthermore, we summarized the literature on characteristics of genotype and phenotype of *UBAP1* in reported HSP patients.

ESCRT-I appears as the stalk-shaped heterotetramer composed of TSG101, VPS28, VPS37, and the mutually exclusive UBAP1 or MVB12. There are multiple isoforms of VPS37(A-D), UBAP1, and MVB12A/B in mammalian cells. The UBAP1 and VPS37A are crucial to binding ubiquitin to function in endosomal sorting. UBAP1 binds the ubiquitylated cargoes through its UBA domain (Stefani et al., 2011), while VPS37A recruited the ESCRT machinery for membrane scission and closure of the phagophore (Takahashi et al., 2019). Until now, *VPS37A* (SPG53) and *UBAP1* have been reported as causative genes for HSP. However, HSPs caused by the mutations in two genes showed different inheritance patterns and phenotypes. The homozygous recessive mutation p. Lys382Asn in the *VPS37A* led to complex HSP, including lower extremity spasticity, cognitive and speech delays (Zivony-Elboum et al., 2012), and HSPs caused by *UBAP1* mutations were inherited in a dominant pattern and presented with the pure or complex form. These findings highlighted that distinct functions of subunits in the ESCRT-I complex contributed to genetic heterogeneity in the pathogenesis of HSP.

It was noteworthy that the reported patients were caused by heterozygous truncating mutations in *UBAP1* which were clustered in the exon 4, especially p. Lys143fs affects 19.3% of HSP patients. All mutations resulted in the deletion of the UBA domain at the C-terminus of UBAP1 but retained the UMA domain at the N-terminus. *In vitro* studies found that overexpression of the N-terminal fragment of UBAP1 containing the UMA domain was capable of forming a multimeric ESCRT-I complex with TSG101, VPS28, and VPS37A, but significantly inhibited the viral budding release and had a dominant-negative effect (Stefani et al., 2011; Agromayor et al., 2012). Moreover, the truncated UBAP1 caused by p. Leu121Profs*18 (Farazi Fard et al., 2019) and p. Lys143fs (Lin et al., 2019), were also found to be incorporated into the ESCRT-I complex but failed to bind the ubiquitinated cargoes. Therefore, the results strongly suggested that the truncating mutations in *UBAP1* are responsible for the pathological process of HSP by a dominant-negative effect on wild-type UBAP1 protein, and then blocked MVB sorting of ubiquitinated proteins in neurons.

Urinary bladder dysfunction is the common complication of HSP with an estimated prevalence of more than 70% in HSP patients (Braschinsky et al., 2010; Fourtassi et al., 2012; Joussain et al., 2019), but shows clinical heterogeneity. However, the relationship between the occurrence of urinary dysfunction and the HSP causative genes remains unclear. For instance, the presence and absence of *SPAST* mutations were not associated with urinary symptoms in HSP patients (Braschinsky et al., 2010). About 25% of *UBAP1*-mutation carriers had urinary bladder dysfunction (Figure 3D). The proband in our study had severe urinary incontinence, whereas other patients in the family did not exhibit the similar symptom. This suggested that even within the same family urinary symptom varied between individuals who shared precisely the same mutation and that there might be no obvious correlation between urinary dysfunction and HSP pathogenic mutations. Moreover, we noticed that the presence of suspected dermoid cyst at the level of the lumbar 4–5 paravertebral segment in the proband might be associated with the development of urinary incontinence. However, the correlation needs to be studied in more clinical cases. It was occasionally reported that spinal cord neoplasms were found in HSP patients, such as intradural extramedullary schwannoma and intramedullary ependymoma (Shin et al., 2020). Recent studies showed that *UBAP1* played a role in tumor development. The expression of *UBAP1* was also significantly decreased in nasopharyngeal carcinoma tissues (Xiao et al., 2006). *UBAP1* affected breast cancer progression by interacting with the long non-coding RNA gene XIST/miR-362-5p axis (Liu et al., 2021). The specific molecular mechanism of *UBAP1* in dermoid cysts and tumors of the nervous system needs to be further studied.

The penetrance of the HSP pathogenic genotype is thought to be related to the age of onset (AO). The AO of patients ranges from infants to the elderly depending on the difference in causative genes, the nature of mutations, and the ethnicity (Elsayed et al., 2021). The most common *SPAST*-related dominant HSP was predominantly adult-onset (average AO was 24.79 years) (Erfanian Omidvar et al., 2021), in contrast, we found that *UBAP1*-related HSP was predominantly children or adolescents (average AO was 10.71 years). Our results also showed that the AO was about 2.64 years younger in European patients with *UBPA1* mutations than in Asian patients, which was consistent with the results by Wang et al., (2020). The difference in AO among patients with different ethnicities was probably related to various variants in modifier genes. It has been found that variants had distinct population genetic characteristics, compared with pathogenic mutations (Sun et al., 2021). The variants in modifier genes also contributed to the differences in the AO of HSP patients. Examples included the patients who carried both *SPAST* and *DPY30* deletion had a significantly younger age at onset than patients with other mutations that were also expected to cause HSP (Newton et al., 2018). Likewise, our findings suggested that different genetic backgrounds were associated with heterogeneity in the AO in patients with *UBAP1*-related HSP.

In summary, we presented a family from north-western China with a novel pathogenic variant (c.279delG) in *UBAP1*. The affected members in the family had pure or complex HSP phenotypes, and only the proband had the symptoms of urinary incontinence and dermoid cyst at the level of L4–L5. However, the correlation between UBAP1 and dermoid cyst development requires further study in the future. In addition, by the literature summary, we analyzed the mutation spectrum and phenotypic properties of *UBAP1*-related HSP. Our results contribute to genetic diagnosis and counseling of HSP syndrome. A considerable effort is still needed to reveal the molecular mechanism of *UBAP1*, thus finding therapeutic targets to limit the detrimental effects of mutations on neuronal function.

## Data Availability

The datasets for this article are not publicly available due to concerns regarding participant/patient anonymity. Requests to access the datasets should be directed to the corresponding author.
