# Supplementary material for: A novel mutation in the UBAP1 gene causing hereditary spastic paraplegia: A case report and overview of the genotype-phenotype correlation
Source: Front Genet. 2022 Jul 14;13:936292. doi: 10.3389/fgene.2022.936292 (PMC9344137; doi:10.3389/fgene.2022.936292)
Supplement: Supplementary file 1 [file DataSheet2.docx]

**Supplemental Figure**

**
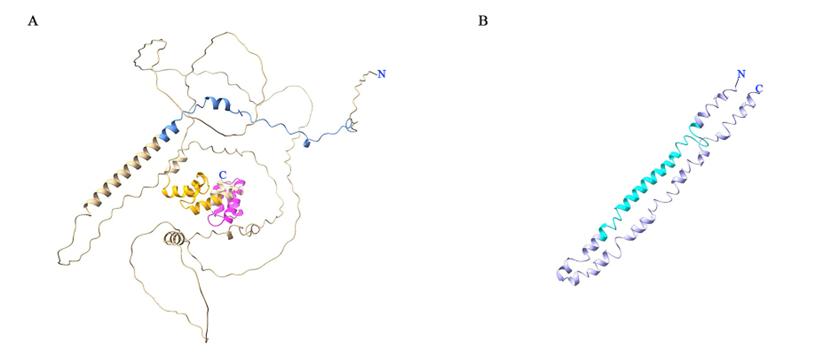
**

**Figure S1. Three-dimensional(3D) structures of the UBAP1 wild-type protein and the identified truncating protein.** (A) The 3D protein structure of the wild-type UBAP1 (AF-Q9NZ09-F1-model_v2 model template). The blue, yellow and pink represent the UMA domain, UBA-1 and UBA-2 domain of UBAP1 protein, respectively. (B) the UBAP1 c.437dupG (NM_016525) resulted in the truncating mutant (p.Ser146ArgfsTer13), and its protein 3D structure was presented after modeling by I-TASSER. The cyan represents the UMA domain in mutant protein. UMA： UBAP1-MVB12-associated domain; UBA: ubiquitin associated domains.

**Supplemental Figure2**


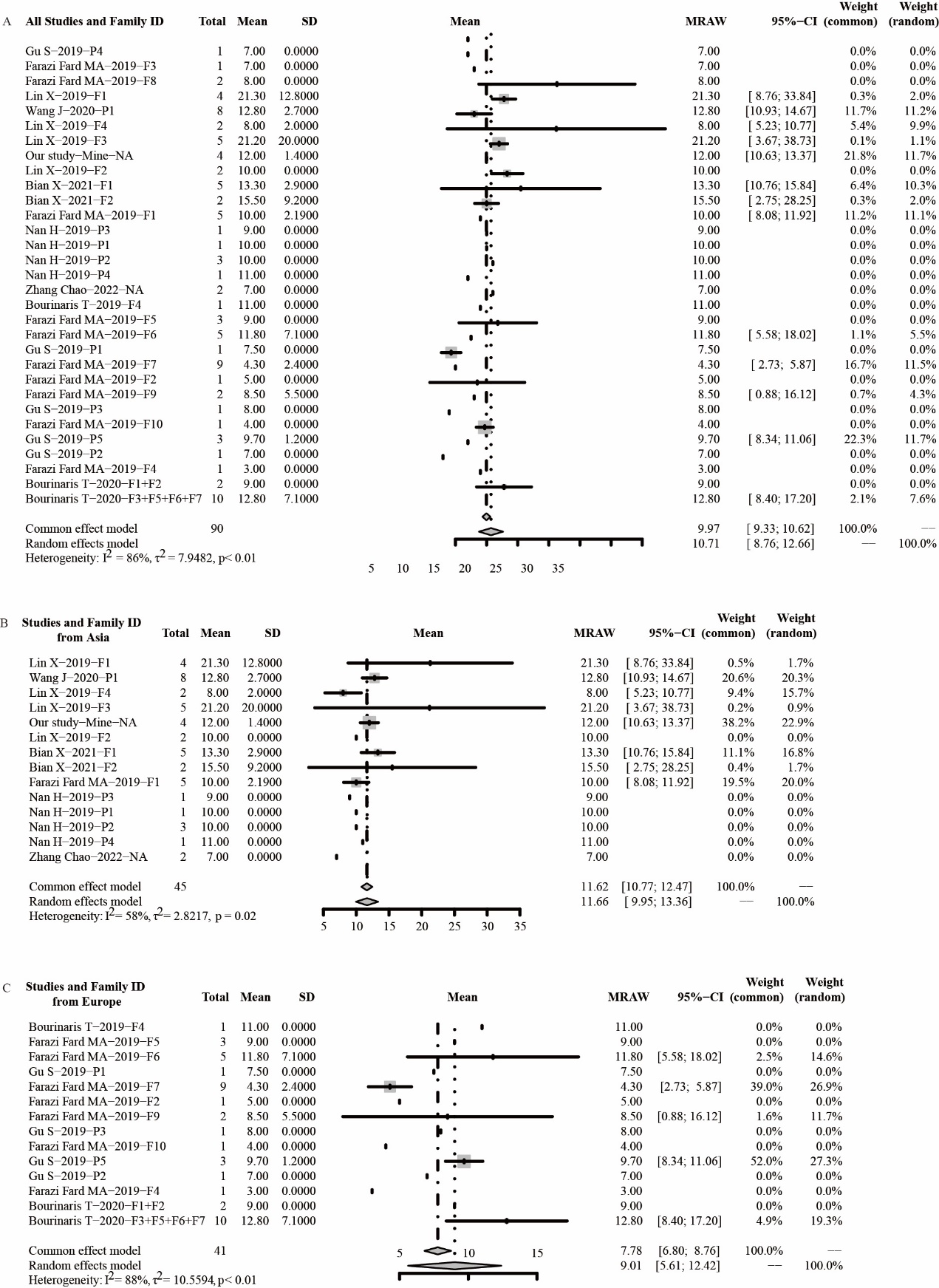


**Figure S2.** **Mean age of onset in *UBAP1*-related HSP patients.** Meta-analysis was used to compare the difference of mean AO. All of included studies(A) were grouped into Asian (B) and European (C). For each study, the black squares are the observed AO and the grey diamonds represent the AO from the fixed-effects model and the random-effects model meta-analysis, respectively. In random effects model, the mean AO of patients was 10.71(95%CI:8.76-12.66). Moreover, the European patients was younger [9.01(95%CI:5.61-12.42)] than Asian patients [11.66(95%CI:9.95-13.36)]. AO: age of onset; CI: confidence interval.
